# Differential Cytotoxicity of MEX: a Component of Neem Oil Whose Action Is Exerted at the Cell Membrane Level

**DOI:** 10.3390/molecules14010122

**Published:** 2008-12-31

**Authors:** Francesca Ricci, Valerio Berardi, Gianfranco Risuleo

**Affiliations:** Dipartimento di Genetica e Biologia Molecolare, Sapienza Università di Roma. P.le Aldo Moro, 5 – 00185 Roma, Italy; E-mails: f.ricci@uniroma1.it (F. R.), valerio.berardi@gmail.com (V. B.)

**Keywords:** Neem oil, MEX, Cytoxicity, Lipo-peroxidation, Cell Membrane.

## Abstract

Neem oil is obtained from the seeds of the tree *Azadirachta indica*. Its chemical composition is very complex, being rich in terpenoids and limonoids, as well as volatile sulphur modified compounds. This work focused on the evaluation of a component of the whole Neem oil obtained by methanolic extraction and defined as MEX. Cytotoxicity was assessed on two different cell populations: a stabilized murine fibroblast line (3T6) and a tumor cell line (HeLa). The data presented here suggest a differential sensitivity of these two populations, the tumor line exhibiting a significantly higher sensitivity to MEX. The data strongly suggest that its toxic target is the cell membrane. In addition the results presented here imply that MEX may contain one or more agents that could find a potential use in anti-proliferative therapy.

## Introduction

Neem oil is a natural mixture of biological interest obtained from the seeds of the tree *Azadirachta indica* (A. Juss), commonly known as the Neem tree. This oil is of common usage in popular medicine and is still considered in India as the “village pharmacy” since its fruit, leaves, bark and roots contain compounds with several biological properties: reports exist on the ability of Neem extracts to combat fungal infections, inflammation as well as viral and bacterial infections [1]. In addition, leaf extracts of *A. indica* show potential antitumor activity, since they protect from tumor induction in a rat model of gastric cancer [2]. Therefore this natural mixture is very interesting for its potential biotechnological applications.

Neem oil has a very heterogeneous composition, which may vary depending upon the habitat and the environmental situation where the plant grows. In any case the oil contains high levels of terpenoids, limonoids, volatile compounds and sulphur modified fatty substances [3]. The most studied component of Neem oil is azadirachtin, a tetra-nor-triterpenoid compound very active in pest and insect control. In our laboratory we prepared a methanolic extract (MEX) of the whole Neem oil, deprived of the terpenoid/limonoid moiety and tested the cytotoxic activity of this azadirachtin-free extract. Our conclusion was that the cytotoxic effect exhibited by MEX is not attributable to azadirachtin but rather to other bio-active molecules [4]. 

One of the research lines developed in our laboratory is the study of the biological activity of different natural compounds [4,5,6,7]. In particular, we recently evaluated the effects of MEX in cultured mouse fibroblasts where we observed a cytotoxic effect induced by this methanolic extract obtained from the whole Neem oil. Our results indicate that MEX might cause cell death via the activation of apoptotic pathway following significant membrane damage [4,5,6,7]. In this report we address two different questions, *i.e.*: 1. Investigating whether MEX has differential cytotoxic action on tumor cells as compared to normal ones and: 2. Elucidating the mode of apoptosis.

The results discussed in this work show that HeLa tumor cells are sensitive to a lower dosage of MEX as compared to stabilized mouse fibroblasts 3T6. Also, the cell membrane appears to be the main target of the extract as shown by lipoperoxidation assays: this observation, in particular, validates and expands former results published by our laboratory [7].

## Results and Discussion

### Effect of MEX on the cell viability: differential sensitivity of 3T6 and HeLa cells

**Figure 1:**
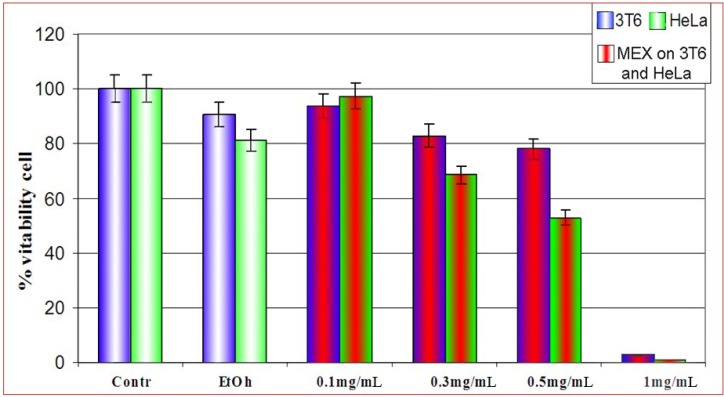
Vitality of 3T6 cell (blue bars) compared to HeLa cells (green bars) after treatment with MEX at different concentrations (central red area). Cell survival is reported as percent with respect to untreated cultures. All samples were in 2% ethanol (final concentration) except for the first two bars to the left where ethanol was absent. Error bars represent the standard error of the mean (mean ± SEM).

In a first series of assays we evaluated the cytotoxicity of MEX both on 3T6 and HeLa cells by the MTT assay [8]. Cells were treated with four different concentrations of extract (Figure 1 and legend, for details). MEX was diluted with ethanol prior to use. At the experimental concentration (2% v/v) this solvent was not detrimental for cell survival since a modest effect on viability as compared to ethanol-free cultures is monitored. Treatment at relatively low concentrations of MEX (0.1 and 0.3 mg/mL) is not dramatically cytotoxic, while viability is strongly reduced at higher concentrations (1.0 mg/mL) where almost the whole cell population fails to survive to the administration of MEX. Analogous treatments on HeLa cells, showed a higher toxicity of MEX as compared to 3T6 in the same experimental conditions. In all subsequent experiments, treatments were performed for 24 hours at 0.5 mg/mL of MEX since this concentration is not too drastic for cells but causes a significant loss of vitality.

The apparently higher sensitivity of HeLa cells to MEX was further investigated by a different approach. The rationale of this experiment was that, due the intrinsically different nature and physiology of the two cell types, a differential sensitivity to MEX might exist.

**Table 1:** Schematic summary of cell death induced by ethanol and Mex on 3T6 and HeLa cells. Treatment with MEX was continued for 24 hrs at a concentration of 0.5 mg/mL. Three independent experiments were performed and results are shown as ± standard deviation (mean ± SD).

3T6 Cells in 2% EtOH			HeLa Cells in 2% EtOH		
Sample	Vital	Dead	Sample	Vital	Dead
1	72±2	6±1	1	75±3	7±1
2	74±2	7±1	2	88±4	10±1
3	81±3	9±2	3	100±2	14±3
Average cell mortality 8.7%			Average cell mortality 9.1%		
MEX-treated 3T6 Cells			MEX-treated HeLa Cells		
Sample	Vital	Dead	Sample	Vital	Dead
1	56±2	12±1	1	36±3	20±2
2	60±1	15±1	2	54±2	42±1
3	48±2	12±2	3	57±2	39±1
Average cell mortality 19.2%			Average cell mortality 40%		

Therefore this hypothesis was further evaluated by vital cell count after Trypan Blue staining. An identical number of the two cell types was plated, MEX was added and the cells were counted after 24 hours of treatment. Results are shown in Table 1: a higher sensitivity of HeLa cells is evident since treatment with MEX causes two-fold higher cell mortality in HeLa as compared to 3T6 cells.

### Selective cytotoxicity can be demonstrated by PCR

**Figure 2:**
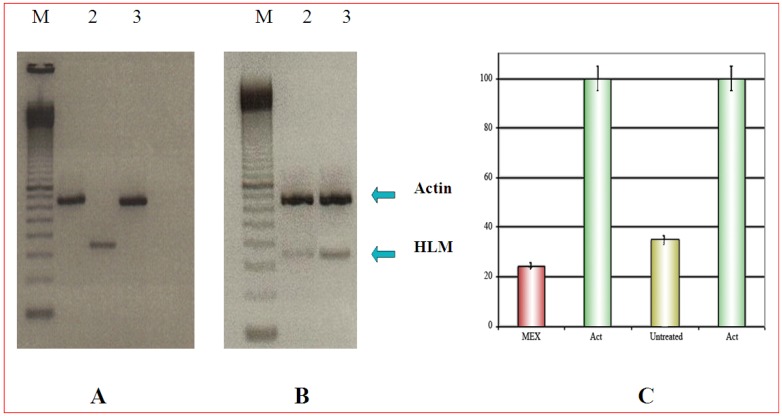
A) Expression of the HLM gene (lane 2) which is absent in 3T6 cells (lane 3). The upper band is the internal reference standard (actin) in HeLa (lane 1) and in 3T6 (lane 3). B) Amplification of the HLM gene in co-cultured 3T6 and Hela cells. Upper band as in Figure 3A. M is a commercial molecular weight marker (100 bp multimer). C) Quantitative analysis of the amplification products reported in B) normalized to the actin amplification product. Error bars (panel C) represent the standard error of the mean (mean ± SEM).

In this experiment 3T6 and HeLa cells were co-cultured. Duplication time of 3T6 is about 1.5 to 2-fold shorter than that of Hela cells. Therefore, different amounts of the two cell types were plated: in this way, at the end of the treatment with MEX, an identical number of cells could be harvested from the same mixed culture. Furthermore, parallel cultures of both cell types were grown separately and, at the end of the experiment, they were trypsinized and counted. In all experiments cell count was never different by more than 5% (data not shown). The analysis by RT-PCR, demonstrated that HLM, a tumor specific protein, as expected, is expressed only in HeLa cells cultures (Figure 2A, lane 2). In the case of co-cultured cells, 5 x 10^5^ 3T6 were plated along with 8 x 10^5^ Hela cells (time 0 of the experiment). The respective number of cells was 25 x 10^5^ 3T6 and 26 x 10^5^ HeLa at harvest time. The result of Figure 2B, shows a fainter band for HLM is after treatment with MEX while the actin band shows the same intensity. This supports the idea that MEX is selectively toxic for tumor cells (Figure 2B, lane 2). 

### The cell membrane is the main target of MEX and the oxidative damage is abolished by the presence of antioxidants.

Treatment with MEX, as already demonstrated in our laboratory by a biophysical approach, alters the structure of the plasma-membrane [7], therefore the level of membrane lipoperoxidation was evaluated since this phenomenon is considered a very good indicator of oxidative stress and membrane damage both in cell cultures and in fresh tissues [9, 10].

**Figure 3:**
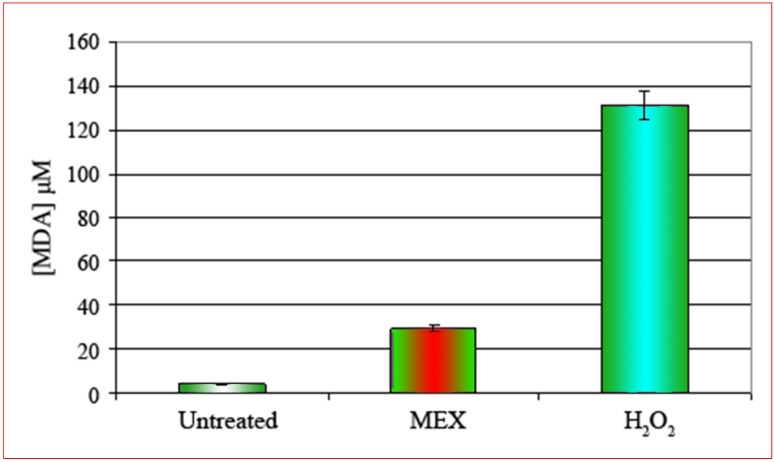
Concentration of malonaldialdehyde [MDA, μM] after treatment with MEX (central bar). MDA concentration is about 25-fold higher as compared to untreated controls (left bar). The effect of treatment with H_2_O_2_ is shown by the right bar. Error bars represent the standard error of the (mean ± SEM).

Extracts were prepared from cells treated according to the standard protocol (24 hours MEX at 0.5 mg/mL) and the concentration of malonaldialdehyde (MDA) was quantitatively measured by spectrophotometry. The positive control was represented by cells treated with hydrogen peroxide (5 minutes at 0.2% final concentration) which is a well known inducer of oxidative stress and consequent apoptosis [11,12,13]. The result shows (Figure 3) that in control cells a very limited, if any, production of MDA occurs. On the contrary the intracellular concentration of MDA increases significantly after treatment with MEX and this is a direct evidence of oxidative stress. In the light of this result we can reasonably conclude that apoptosis caused by MEX is attributable to oxidative stress with consequent structural and functional alteration of the plasma membrane.

**Figure 4:**
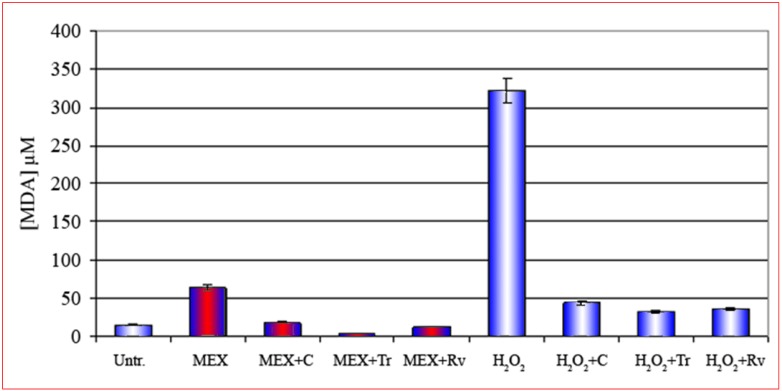
Reduction of the production of MDA in 3t6 cells after treatment with MEX (red central area) in the presence of three different antioxidant curcumin (C), trolox (Tr) and resveratrol (Rv) shown respectively in the third, fourth and fifth bar. These compounds reduce drastically the oxidative stress also after treatment with H_2_O_2_ in the absence of MEX (bars with white central area). Error bars represent the standard error of the standard of the mean (mean ± SEM).

This conclusion is further corroborated by the results presented in Figure 4. As a matter of fact, the treatment with MEX in presence of three compounds known for their action as peroxide scavengers and/or antioxidants such as curcumin (third bar from the left), Trolox® (fourth bar) and resveratrol (fifth bar) [14,15,16,17,18], strongly reduce the intracellular formation of MDA. This result, combined the lipoperoxidation data, indicates that the methanolic extract induces an oxidative stress at membrane level which eventually leads to apoptotic death.

Neem oil is a natural complex mixture endowed of diverse biological properties: for centuries it has been used in Ayurvedic and popular medicine. However a few recent scientific works investigated the potential of this natural mixture and of purified compounds in the control of virus and cell proliferation. In this work we evaluate the differential cytotoxic activity of the methanolic extract MEX towards normal and tumor cells. The negative control of cell proliferation exerted by MEX was already investigated in this laboratory [4] and, in addition, the cytotoxic effect is more pronounced on tumor cells as evidenced by the data presented in this work.

All treatments with MEX were performed at a concentration which is not dramatically toxic. As a matter of fact, survival of about 50% of the cell population is observed after 24 hour of exposure to the mixture. Thus, one can investigate the effects of MEX at cellular/molecular level, since a significant portion of the cell population in still viable. Also, it is worth noting that an evident threshold effect exists between 0.3 and 0.5 mg/mL: as a matter of fact at the lower concentration 3T6 cells seem to be unaffected while HeLa cells show a more pronounced mortality. 

The differential activity of MEX on tumor cells was evidenced by MTT evaluation of cell survival as well as by vital cell counts after Trypan blue staining. The apparent discrepancy in survival rate measured by MMT and Trypan blue vital cell count is very likely due to the difference in the approach: namely, MTT permits a specific and quantitative evaluation of the mitochondrial damage while Trypan blue reveals the actual number of dead cells, since these are no longer able to eliminate the dye from the cytoplasm. Therefore Trypan blue acts at a more general level. The differential mortality was validated also by experiments where HeLa cells were co-cultured with 3T6 mouse fibroblasts. The duplication time for 3T6 and Hela is different, therefore we set up adequate plating conditions to have about the same number of cells at the end of the experiment. In any case, in each experiment, parallel plates were grown and treated in same conditions but seeded with only one cell type. At the end of each experiment cells were trypsinized and counted to assure that their number was comparable: the final number of cells in no case differed more than 5%. The results of these experiments demonstrated by RT PCR that the level of the mRNA for the HLM gene, which is expressed specifically in tumor cells and is involved in the generation of metastases [19], is significantly reduced after treatment with MEX. This data is qualitative but, in any case, suggests that MEX could have a potential use as anti-proliferative drugs. The active purified component is yet to be identified although work is in progress in our laboratory (Ricci, Nardone, Risuleo, manuscript in preparation). However, one could speculate that a single purified component may not be as efficient as the MEX fraction since synergic effects of different components present in the mixture may occur (Ricci, unpublished observations). 

Cell death follows two different pathways: necrosis and apoptosis but the two mechanisms are not mutually exclusive. In a previous paper we presented data suggesting that MEX induces cell death by apoptosis [4]. In addition, data from our laboratory, obtained by a biophysical approach, strongly suggest that MEX alters the dielectric properties of the membrane thus inducing extensive damage [7]. In this work we demonstrate, by a specific and quantitative lipo-peroxidation assay, that the main target of MEX is indeed the cell membrane. These data were obtained measuring the intracellular concentration of MDA which is produced as a consequence of oxidative stress at lipid level. MDA is a very reactive dihaldeyde [9, 20] which forms stable covalent bonds inducing structural alterations of biopolymers in general and of phospholipids in particular. The negligible amount of MDA found also in untreated sample must considered “physiological” since it derives, very likely, from not fully functional cells present in the controls. The oxidative stress was abolished if the treatment with MEX was performed in the presence of scavengers of reactive oxygen species such as trolox, a water soluble analogous vitamin E [14,15,16], or known antioxidants such as curcumin [17] and resveratrol [18]. It is known that the cross-links phospholidid-MDA-phospholipid cause, eventually, an increased membrane fluidity [21] which ends in its structural damage [7]. Furthermore, the abolition of the oxidative damage by curcumin implies that the endoplasmic reticulum, as demonstrated in other experimental models [17], is involved in the apoptotic process activated by MEX. The data presented here refer to 3T6 cells only, but analogous results were obtained with Hela cells (data not shown).

## Conclusions

In conclusion, the cytotoxicity of MEX is attributable to the drastic structural alterations of the plasma membrane which involves the destruction of the membrane lipids and consequent loss of function. This may also explain the reason for the differential toxicity exerted by MEX on tumor cells: it is known in fact, that tumor transformation induces modifications of plasma membrane lipid bi-layer and an increased level of lipid phosphorylation. In the light of these results it could be envisaged a possible use of Neem components as anti-proliferative agents.

## Experimental

*Cell cultures:* both 3T6 and HeLa cells were grown in high glucose DMEM, supplemented with newborn bovine serum (10% final concentration) glutamine (50 mM) and penicillin-streptomycin (10000 U/mL). Growth temperature was 37 °C in controlled humidity at 5% CO_2_. Cells were routinely passaged every third day. 

*Preparation of MEX:* Whole Neem oil was supplied by Trifoglio-MR GmgH (Lahnau – Germany). This material was used throughout the work since its formulation is consistent, thus providing reproducible results. The Methanolic Extract (MEX) was obtained by six consecutive methanol extractions (twice the volume of the whole oil each time). The extract was subsequently freeze-dried *in vacuo* and the pellet thus obtained was dissolved in ethanol in a stock solution at a concentration of 100 mg/mL: for details on the procedure see [4]. 

*Cell viability:* cell viability was assessed by the colorimetric MTT assay [8]. Absorbance was measured at 570 nm to obtain a standard cell count. The number of cells surviving to the treatment with MEX was also evaluated by vital cell count in Trypan Blue in a Burker chamber.

*RNA extraction and RT-PCR conditions:* total RNA purification was performed by the Nucleospin RNA extraction kit (Macherey-Nagel). The cDNA synthesis was performed using 1 μg of total RNA as template and 200 units of MMLV reverse transcriptase (Invitrogen). Amplification by PCR was performed using a Geneamp 2400 (Applied Biosystems), and ExTaq DNA polymerase (Takara). RT-PCR was performed in the following conditions: annealing temperature was 63.5 °C for the actin and 63.9° C for the HLM primers, respectively. The thermal cycler was set at 72 °C for 5 minutes with intervals at 60 °C (30 seconds), denaturation was at 94 °C, a total number of 25 cycles was performed. Below the forward and reverse primers for actin and HLM are reported:

Act fw: 5’ CGGGGTCTTTGTCTGAGC 3’, Act rv: 5’ CACGATTGGGGATAAAGGAA 3’
HLM fw: 5’GTGCACTTGGAGGAAACCAT3’, HLM rv: 5’ACTCGCCTCTTGACTTTGGA3’

**Figure 5:**
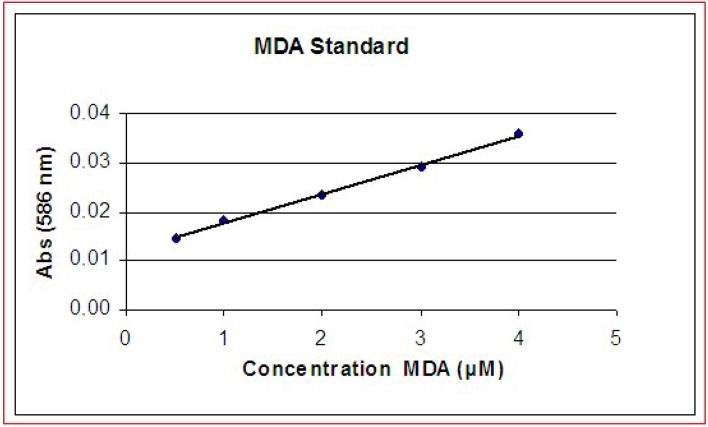
Production of MDA plotted *vs.* absorbance with the formation of a standard reference curve.

*Evaluation of the cytoplasm membrane stress*: lipid peroxidation is a commonly accepted diagnostic and well established biochemical mechanism of cellular damage in both plants and animals. Evaluation of lipid peroxidation is a good indicator of oxidative stress in cultured cells and in tissues [22,23,24]. To determine the oxidative stress at membrane level we used a quantitative assay based on the intracellular formation of malondialdehyde (MDA) measured by the commercial kit LPO-586 (Oxis Health Research Products Portland, Or. USA. This assay is based on the reaction of the chromophore, N-methyl-2-phenylindole (NMP) reacts with MDA and 4-hydroxyalkenals after incubation at 45 °C. A single MDA molecule reacts with NMP molecules generating a stable chromophore whose absorbance can be spectrophotometrically measured at a 586 nm. Absorbance values can be directly converted in molar concentration of MDA and an example of standard curve is provided in Figure 5.

*Statistical analysis:* data are presented as mean values of at least 3 independent experiments ± standard deviation (mean ± SD) (Table 1) or ± standard error (mean ± SEM) (see captions of each figure).

## References

[1] Subapriya R., Nagini S. (2005). Medicinal properties of neem leaves: a review. Curr. Med. Chem. Anticancer Agent.

[2] Subapriya R., Nagini S. (2003). Ethanolic neem leaf extract protects against N-methyl-N'-nitro-N-nitrosoguanidine-induced gastric carcinogenesis in Wistar rats. Asian Pac. J. Cancer Prev..

[3] Schmutterer H. (2002). The neem tree and other meliaceous plants.

[4] Di Ilio V., Pasquariello N., van der Esch S.A., Cristofaro M., Scarsella G., Risuleo G. (2006). Cytotoxic and antiproliferative effects induced by a non terpenoid polar extract of *A. indica* seeds on 3T6 murine fibroblasts in culture. Mol. Cell. Biochem..

[5] Iacoangeli A., Melucci-Vigo G., Risuleo G. (2000). Mechanism of the inhibition of murine polyomavirus DNA replication induced by the ionophore monensin. Biochimie.

[6] Campanella L., Delfini M., Ercole P., Iacoangeli A., Risuleo G. (2002). Molecular characterization and action of usnic acid: a drug that inhibits proliferation of mouse polyomavirus *in vitro* and its main target is RNA transcription. Biochimie.

[7] Bonincontro A., Di Ilio V., Pedata O., Risuleo G. (2007). Dielectric properties of the plasma membrane of cultured murine fibroblasts treated with a nonterpenoid extract of *Azadirachta indica* seeds. J. Membr. Biol..

[8] Mosmann T. (1983). Rapid colorimetric assay for cellular grow and survival: application to proliferation and cytotoxixity assay. J. Immunol. Methods.

[9] Chancerelle Y., Kergonou J.F. (1995). Immunologic relevance of malonic dialdehyde. Ann. Pharm. Fr..

[10] Petit E., Divoux D., Chancerelle Y., Kergonou J.F., Nouvelot A. (1995). Immunological approach to investigating membrane cell damages induced by lipoperoxidative stress. Application to far UV-irradiated erythrocytes. Biol. Trace Elem. Res..

[11] Shimizu S., Umezawa K., Takada M., Arber N., Imoto M. (1998). Induction of hydrogen peroxide production and Bax expression by caspase-3(-like) proteases in tyrosine kinase inhibitor-induced apoptosis in human small cell lung carcinoma cells. Exp. Cell. Res..

[12] Yang H.W., Hwang K.J., Kwon H.C., Kim H.S., Choi K.W., Oh K.S. (1998). Detection of reactive oxygen species (ROS) and apoptosis in human fragmented embryos. Hum. Reprod..

[13] Yamamoto H., Ozaki T., Nakanishi M., Kikuchi H., Yoshida K., Horie H., Kuwano H, Nakagawara A. (2007). Oxidative stress induces p53-dependent apoptosis in hepatoblastoma cell through its nuclear translocation. Genes Cells.

[14] Bonincontro A., Iacoangeli A., Melucci-Vigo G., Risuleo G. (1997). Apoptosis dependent decrease of the intramembrane ion traffic in cultured mousefibroblasts shown by conductivity dispersion. Biosci. Rep..

[15] Cristofanilli M, Pescosolido N., Risuleo G., Scarsella G. (2001). A murine cell culture model for post-trabeculectomy anfibrotic treatment:Induction of apoptosis by Cyclosporin. Acta Ophthalmol. Scand..

[16] Calandrella N., Scarsella G., Pescosolido N., Risuleo G. (2007). Degenerative and apoptotic events at retinal and optic nerve level after experimental induction of ocular hypertension. Mol. Cell. Biochem..

[17] Bakhshi J., Weinstein L., Poksay K.S., Nishinaga B., Bredesen D.E., Rao R.V. (2008). Coupling endoplasmic reticulum stress to the cell death program in mouse melanoma cells: effect of curcumin. Apoptosis.

[18] de la Lastra C.A., Villegas I. (2007). Resveratrol as an antioxidant and pro-oxidant agent: mechanisms and clinical implications. Biochem. Soc. Trans..

[19] Fournier M.V., Guimaraes da Costa F., Paschoal M.E., Ronco L.V., Carvalho M.G., Pardee A.B. (1999). Identification of a gene encoding a human oxysterol-binding protein-homologue: a potential general molecular marker for blood dissemination of solid tumors. Cancer Res..

[20] Draper H.H., Hadley M. (1990). A review of recent studies on the metabolism of exogenous and endogenous malondialdehyde. Xenobiotica.

[21] Cazzola R., Russo-Volpe S., Cervato G., Cestaro B. (2003). Biochemical assessments of oxidative stress, erythrocyte membrane fluidity and antioxidant status in professional soccer players and sedentary controls. Eur. J. Clin. Invest..

[22] Esterbauer H., Schaur R.J., Zollner H. (1991). Chemistry and biochemistry of 4-hydroxynonenal, malonaldialdehyde and related aldehydes. Free Rad. Biol. Med..

[23] Montilla-López P., Muñoz-Agueda M.C., Feijóo López M., Muñoz-Castañeda J.R., Bujalance-Arenas I., Túnez-Fiñana I. (2002). Comparison of melatonin versus vitamin C on oxidative stress and antioxidant enzyme activity in Alzheimer's disease induced by okadaic acid in neuroblastoma cells. Eur. J. Pharmacol..

[24] Nair U., Bartsch H., Nair J. (2007). Lipid peroxidation-induced DNA damage in cancer-prone inflammatory diseases: a review of published adduct types and levels in humans. Free Rad. Biol. Med..

